# Dual effect of *Lutzomyia longipalpis* saliva on *Leishmania braziliensis* infection is mediated by distinct saliva-induced cellular recruitment into BALB/c mice ear

**DOI:** 10.1186/1471-2180-13-102

**Published:** 2013-05-08

**Authors:** Vanessa Carregaro, Diego Luis Costa, Claudia Brodskyn, Aldina Maria Barral, Manuel Barral-Netto, Fernando Q Cunha, João Santana Silva

**Affiliations:** 1 Department of Biochemistry and Immunology, School of Medicine of Ribeirão Preto, University of São Paulo, Av Bandeirantes, 3900. Ribeirão Preto, São Paulo, Brazil; 2 Department of Pharmacology, School of Medicine of Ribeirão Preto, University of São Paulo, São Paulo, Brazil; 3 Centro de Pesquisa Gonçalo Muniz, Fundação Oswaldo Cruz (FIOCRUZ), São Paulo, Brazil; 4 School of Medicine, Federal University of Bahia, Salvador, Brazil; 5 Institute of Immunological Research, Salvador, Brazil

**Keywords:** *Phlebotomines* saliva, *Lutzomyia longipalpis* saliva, *Leishmania braziliensis*, Inflammatory leucocytes, Cytokines, Immunoregulation

## Abstract

**Background:**

*Leishmania* parasites are transmitted to their vertebrate hosts by infected Phlebotomine sand flies during the blood meal of the flies. Sand fly saliva is known to enhance *Leishmania* spp. infection, while pre-exposure to saliva protects mice against parasitic infections. In this study, we investigated the initial inflammatory leucocyte composition induced by one or three inocula of salivary gland extract (SGE) from *Lutzomyia longipalpis* in the presence or absence of *Leishmania braziliensis*.

**Results:**

We demonstrated that inoculating SGE once (SGE-1X) or three times (SGE-3X), which represented a co-inoculation or a pre-exposure to saliva, respectively, resulted in different cellular infiltrate profiles. Whereas SGE-1X led to the recruitment of all leucocytes subtypes including CD4^+^ T cells, CD4^+^CD25^+^ T cells, dendritic cells, macrophages and neutrophils, the immune cell profile in the SGE-3X group differed dramatically, as CD4^+^ T cells, CD4^+^CD25^+^ T cells, dendritic cells, macrophages and neutrophils were decreased and CD8^+^ T cells were increased. The SGE-1X group did not show differences in the ear lesion size; however, the SGE-1X group harbored a higher number of parasites. On the other hand, the SGE-3X group demonstrated a protective effect against parasitic disease, as the parasite burden was lower even in the earlier stages of the infection, a period in which the SGE-1X group presented with larger and more severe lesions. These effects were also reflected in the cytokine profiles of both groups. Whereas the SGE-1X group presented with a substantial increase in IL-10 production, the SGE-3X group showed an increase in IFN-γ production in the draining lymph nodes. Analysis of the inflammatory cell populations present within the ear lesions, the SGE-1X group showed an increase in CD4^+^FOXP3^+^ cells, whereas the CD4^+^FOXP3^+^ population was reduced in the SGE-3X group. Moreover, CD4^+^ T cells and CD8^+^ T cells producing IFN-γ were highly detected in the ears of the SGE-3X mice prior to infection. In addition, upon treatment of SGE-3X mice with anti-IFN-γ monoclonal antibody, we observed a decrease in the protective effect of SGE-3X against *L. braziliensis* infection.

**Conclusions:**

These results indicate that different inocula of *Lutzomyia longipalpis* salivary gland extract can markedly modify the cellular immune response, which is reflected in the pattern of susceptibility or resistance to *Leishmania braziliensis* infection.

## Background

Leishmaniasis is a vector-borne disease transmitted exclusively by sand fly bites [1], during which the host is inoculated with saliva. The saliva has been shown to downregulate the immune response allowing the establishment of successful pathogen infection [2-4]. Co-injection of *Leishmania* and salivary gland homogenates from either *Lutzomyia longipalpis* or *Phlebotomus papatasi* in naïve mice produces a substantial increase in lesion size and parasite burden. The increase in infectivity was associated with the capacity of the saliva to selectively inhibit antigen presentation and nitric oxide (NO) and hydrogen peroxide production thus inhibiting the ability of macrophages to kill the intracellular parasites [5,6]. Furthermore, *Leishmania* vector saliva inhibits the production of protective type 1 cytokines such IL-12 and IFN-γ [7-9], while enhancing the production of interleukin (IL)-10, IL-4, IL-6 and prostaglandin E (PGE)_2_, all of which enhance parasite survival [10-13].

Pre-exposure to saliva or bites from uninfected sand flies can lead to an increase in host resistance to *Leishmania* as a consequence of developing a long-term humoral immune response against the salivary components responsible for pathogen establishment [14]. However, the saliva-induced protection was associated with a delayed–type hypersensitivity (DTH) response and the upregulation of IFN-γ and IL-12 at the site of inoculation [15]. Vaccinating mice against Maxidilan (MAX), the potent salivary vasodilatador from *Lutzomyia longipalpis* sand fly, protected the animal from *L. major* infection by eliciting anti-MAX antibodies and a Th1 immune response [14]. Moreover, mice inoculated with a 15-kDa salivary protein (PpSP15) produced a strong DTH response, which even occurred in B cell knockout mice, suggesting that the cellular immune response against the saliva provided most, if not all, of the protective effect [16]. However, the mechanism responsible for the saliva-induced dual immunity observed in *Leishmania* infections remains unknown.

Cell recruitment is a vital event during inflammation. The cell number and cellular composition soon after an inflammatory stimulus is encountered greatly influences the future responses and the development of an adaptive immune response. Leukocyte recruitment to infected tissue is a crucial event for the control of infections such as leishmaniasis [17,18]. Furthermore, clinical leishmaniasis lesions are associated with an influx of inflammatory cells [19].

Sand fly saliva contains a mixture of pharmacologically active compounds that influence leucocyte migration. *Phlebotomus dubosqi* saliva attracts vertebrate monocytes *in vitro*[20] and *P. papatasi* saliva attracts macrophages and enhances infections by *Leishmania donovani* resulting in an increased parasitic load [21]. *Lutzomyia longipalpis* and *P. papatasi* saliva recruit eosinophils and macrophages through the release of Th2 cytokines and chemokines [13,17,18]. Neutrophils are recruited to the site of *Leishmania* inoculation during the bite of an infected sand fly and prevent parasite surveillance via oxidant- and protease-dependent mechanisms [22]. The co-injection of *L. major* with *Lutzomyia longipalpis* saliva increases the number of CD4^+^CD45RB^low^ T cells within the inoculation site. Undoubtedly, sand fly saliva directly influences the recruitment of leucocytes by altering the adaptive immune response. In the current study, we characterized the distinct cellular composition within BALB/c mouse ears following the inoculation of salivary gland extract (SGE) from *Lutzomyia longipalpis* in association with distinct patterns of resistance or susceptibility to *L. braziliensis* infection.

## Methods

### Mice

Male BALB/c mice weighing 18–22 g were housed in temperature-controlled rooms (22-25°C) with *ad libitum* access to water and food in the animal facility of the Department of Immunology, School of Medicine of Ribeirão Preto, University of São Paulo (Brazil). All experiments were conducted in accordance with NIH guidelines on the welfare of experimental animals, and all experiments were approved by the Ribeirão Preto School of Medicine Ethics Committee.

### Salivary gland extract (SGE)

SGE was prepared from 7- to 10-day-old laboratory-bred, female *Lutzomyia longipalpis* as previously described [23]. Briefly, 50 pairs of salivary glands were dissected under sterile conditions in endotoxin-free PBS, placed in 50 μl of PBS and were kept at −70°C until use. Immediately before use, the glands were disrupted by sonication using a Sonifer 450 homogenizer (Branson, Danbury, Connecticut). Endotoxin levels were evaluated by using the QCL-1000(r) Chromogenic LAL Endpoint Assay kit (Lonza, Switzerland), which revealed negligible levels of endotoxin within the salivary gland supernatants.

### SGE intradermal inoculation

The ear dermis of BALB/c mice was intradermally inoculated with different inoculums of SGE (SGE-1X and SGE-3X). Each inoculum consisted of 0.5 pair of SGE diluted in 10 μL of PBS /ear. SGE-1X group received one single inoculum of SGE and, other group, the mice received SGE-1X plus promastigote forms of *L. braziliensis* (1 × 10^5^). The protocol of immunization with saliva consisted of three inoculums of SGE, with intervals of 10 days among each ones. Alternatively, the mice received three inoculums of SGE being that, in the third one, they also received the infection with parasites*.* The control group, the mice received one injection with 10 uL of PBS. Thus, the groups are: Group PBS = one injection of PBS; Group SGE-1X = one injection of SGE; Group SGE-3X = three injections of SGE; Group PBS/parasite = PBS plus parasite; Group SGE-1X/parasite = SGE-1X plus parasite; Group SGE-3X/parasite = SGE 2X + SGE-1X plus parasite.

### Parasitic, intradermal infection and parasitic burden quantification

*L. braziliensis* was cultured in Schneider (Sigma, Saint Louis, MO, USA) medium supplemented with 20% heat-inactivated fetal calf serum (Cultilab, Campinas, SP, Brazil), 4 mM NaHCO_3_, 100 U/ml penicillin, 100 μg/ml streptomycin (all from Gibco, Grand Island, NY, USA), and 2% v/v male human urine at 25°C. Promastigotes of *L. braziliensis* were isolated from stationary phase cultures (5-6th day of culture), centrifuged at 1540 g at 4°C for 10 min and washed in PBS. Promastigotes of *L. braziliensis* were isolated from stationary phase cultures (5-6th day of culture), centrifuged at 1540 × g at 4°C for 10 min and washed in PBS. The *L. braziliensis* promastigotes (1 × 10^5^) were inoculated intradermally into the ear of mice previously inoculated with SGE (−1X or -3X) or vehicle (PBS) using a 27.5-G needle in a total volume of 10 μl. The development of lesions was monitored by measuring the diameter of the ear lesion with a vernier caliper. To quantify the parasitic burden, the dermal sheets of the infected ears were separated, deposited dermal side down, and then homogenized using a Medimachine (Becton & Dickinson Biosciences, San Diego, CA, USA) tissue grinder in a microfuge tube containing 1000 μl of supplemented Schneider medium (Sigma, Saint Louis, MO, USA) for 4 min. The tissue homogenates were serially diluted in 96-well flat-bottom microtiter plates (Corning Incorporated, NY, USA) containing biphasic medium prepared using 50 μl of NNN medium with 30% of defibrinated rabbit blood and were overlaid with 100 μl of Schneider medium (Sigma). The number of viable parasites in each tissue was determined from the highest dilution at which promastigotes could be grown after 7 days of incubation at 26°C.

### Leucocyte isolation from lesions

To characterize the leucocytes within the inoculation site, the inflammatory cells were recovered as previously described [24]. Briefly, at different time points after intradermal inoculation, ears were collected and incubated at 37°C for one hour in RPMI-1640 medium containing 2 mM L-glutamine, 100 U/ml penicillin, 100 μg/ml streptomycin (all from Gibco, Grand Island, NY, USA) and 500 μg/ml Liberase CI (Roche, Basel, Switzerland). The tissues were processed inside Medcons using a Medimachine (both from BD Biosciences). After processing, the cells were filtered through a 50-μm filter, viability was assessed by trypan blue exclusion, and the cell concentration was determined.

### Flow cytometry

The dermal inflammatory cells were gated based on their characteristic size (FSC) and granularity (SSC), and the T lymphocytes (CD4^+^CD3^+^, CD8^+^CD3^+^and CD4^+^CD25^+^) dendritic cells (CD11c^+^CD11b^+^MHC-II^+^), macrophages (F4/80^+^CD11c^-^MHC-II^+^) and neutrophils (Gr1^+^MHC-II^-^) (BD Biosciences) were identified individually. The isotype controls used were rat IgG2b and rat IgG2a. For regulatory T cell phenotyping, CD4^+^CD25^+^ T cells were stained with anti-FoxP3 antibody conjugated to phycoeritrin (PE) (e-Biosciences). For intracellular staining, the cells were permeabilized using a Cytofix/Cytoperm kit (BD Biosciences) according to the manufacturer’s instructions. For all analyses, the results were compared with the results obtained from cells stained with isotype control antibodies. Cell acquisition was performed using a FACSort flow cytometer. Data were plotted and analyzed using Cell Quest (BD Biosciences) and FlowJo software (Tree Star, Ashland, OR).

### Cytokine release

To assess the influence of SGE on cytokine production, single-cell suspensions of the draining retromaxillar lymph nodes from the SGE-1X-, SGE-3X- or PBS-inoculated mice were prepared aseptically, diluted to a concentration of 2 × 10^6^ cells/ml, and dispensed into 48-well plates in a total volume of 500 μl of complete RPMI-1640 medium with or without 5 × 10^6^ live stationary phase *L. braziliensis* promastigotes. Cell culture supernatants were harvested after 72 hours of culture at 37°C in 5% CO_2_, and the levels of IFN-γ (BD Biosciences) and IL-10 (R&D Systems Minneapolis, MN, USA) were determined by using commercial ELISA kits, according to the manufacturer’s instructions.

### *In vivo* depletion of IFN-γ cytokine

R46A2 hybridoma cells secreting rat IgG1 anti-IFN-γ were used in this study. These cells were grown as ascites in pristine (Sigma)-primed, nude-backcrossed BALB/c mice. R46A2 antibodies were purified from ascitic fluid as described elsewhere [25]. Groups of SGE-3X-inoculated BALB/c mice were given 500 μg of R46A2 mAb or normal rat IgG control antibody intraperitoneally 1 day before infection with *L. braziliensis* (day 1) and were given 250 μg of the respective antibody every 3 days for 3 weeks thereafter.

### Statistical analyses

The data are reported as the mean ± SEM and are representative of two or three independent experiments. The means between different groups were compared by the analysis of variance (ANOVA) followed by the Tukey test for unpaired values. P<0.05 was considered to be statistically significant.

## Results

### Kinetics of the SGE effect in the recruitment of leucocytes to the site of inoculation in BALB/c mice

**Figure 1:**
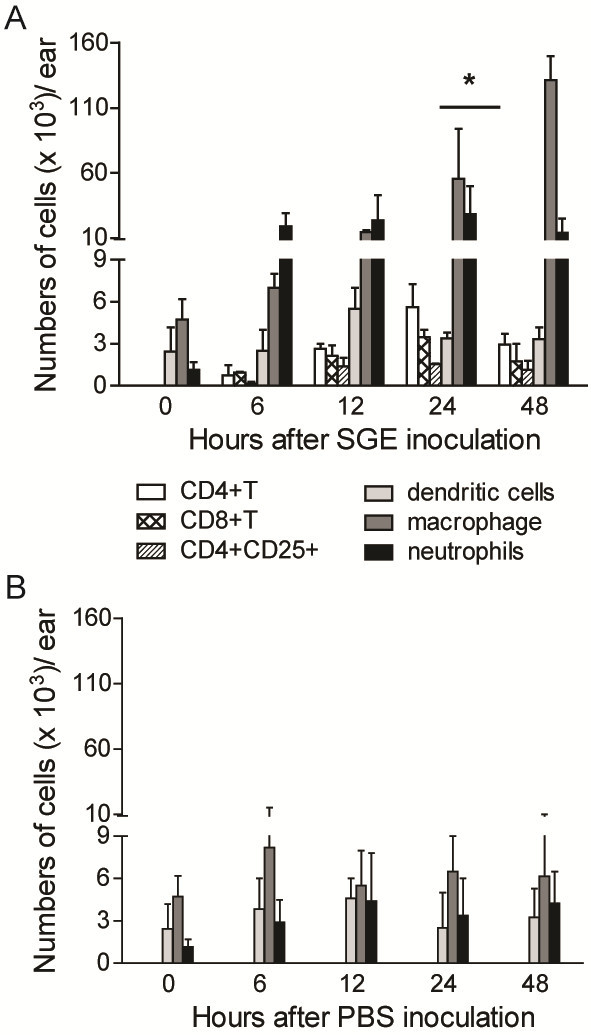
**Kinetics of the inflammatory infiltrate induced by *Lutzomyia longipalpis *saliva at the site of inoculation.** BALB/c mice were inoculated intradermally within the ear dermis with half of the salivary gland extract (SGE) generated from the two salivary glands diluted in 10 μl of PBS **(A)** or a injection with PBS only **(B)**. The leucocytes from three mouse ears/group were obtained at 0, 6, 12, 24 and 48 h after inoculation, and different populations were identified using flow cytometry. The data showed represent the mean ± SEM and are representative of three independent experiments (n = 3). *P < 0.05 compared to 0 hours (naive).

We analyzed the accumulation of leukocytes in the dermis after 0, 6, 12, 24 and 48 hours post-intradermal inoculation of *Lutzomyia longipalpis* salivary gland extract (SGE) (0.5 pair of glands/mouse) into the ears of BALB/c mice. SGE induced neutrophils (GR1^+^MHC-II^–^) cell recruitment 6 hours post-inoculation, which persisted for 48 hours. CD4^+^ T cells, CD8^+^ T cells and CD4^+^CD25^+^ T cells appeared 12 hours post-inoculation and persisted during all period analyzed. Macrophages (F4/80^+^CD11c^-^ MHC-II^+^) cell accumulation was observed 12 hours after inoculation, and dendritic cells (CD11b^+^CD11c^+^MHC-II^+^) levels did not change (Figure 1A).

To determine that the leukocyte migration is SGE-specific and not due damage inflicted by the needle injection, the kinect of leucocyte migration after similar amounts of PBS (10 μL) inoculated into ears of mice was performed. As showed, the amounts of dendritic cells, neutrophils, macrophages in PBS-inoculated mice was similar in all time points analyzed and was comparable that those recovered from naïve ears mice (Figure 1B), confirming the specificity of SGE in the leukocyte recruitment.

### Inflammatory infiltrate after one or three inocula of SGE

**Figure 2:**
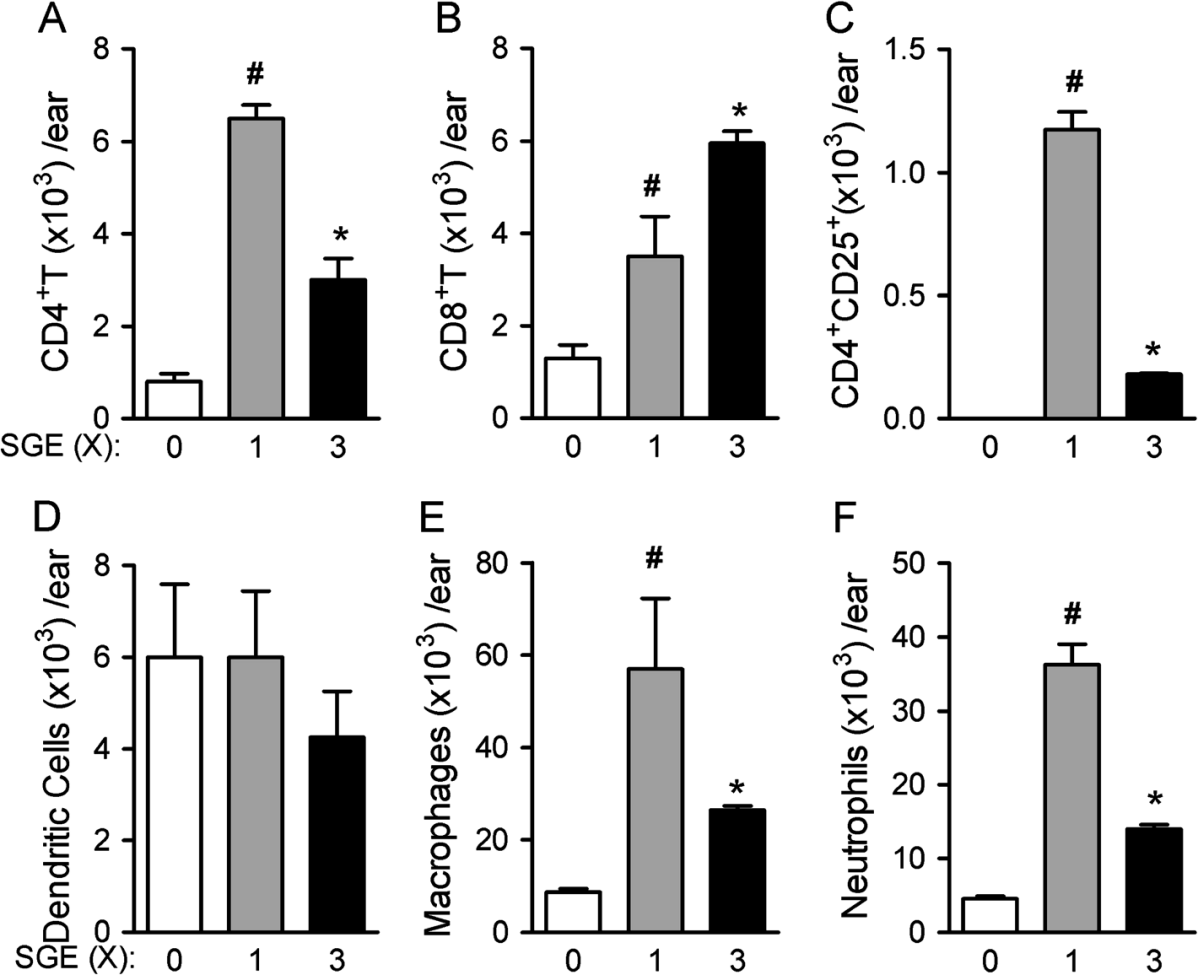
**Comparative analyses of the inflammatory infiltrate into the site of infection after SGE inoculation.** BALB/c mice were inoculated i.d. once (SGE-1X-gray bars) or three times (SGE-3X–black bars) within the ear dermis with SGE (derived from 0.5 pair of salivary glands diluted in 10 μl of PBS/ear) or PBS (10 μl/ear-white bars). The mice were euthanized 24 h later, and ears were harvested for inflammatory infiltrate characterization. The total number of CD4^+^ T cells **(A)**, CD8^+^ T cells **(B)**, CD4^+^CD25^+^ cells **(C);** dendritic cells **(D)**, macrophages **(E)** and neutrophils **(F)** present within the ears were identified by flow cytometry. Data represent the mean ± SEM and are representative of three independent experiments (n = 4). ^#^*P* < 0.05 compared with PBS (control group). ^*^P < 0.05 compared with the SGE-1X group.

Next, we determined whether saliva promotes or protects against leishmaniasis. First, we compared the inflammatory infiltrate after different injections of SGE. BALB/c mice received one or three intradermal ear injections of SGE, and the emigrated leucocytes were analyzed. As a control group, BALB/c mice were inoculated with PBS (time 0). Our results show that the SGE-1X group had an increased recruitment of different subtypes analyzed: CD4^+^ T cells, CD8^+^ T cells, CD4^+^CD25^+^ cells, macrophage and neutrophil (Figure 2). The leukocyte influx into the ears of mice from the SGE-3X group consisted of CD4^+^ T cells, CD4^+^CD25^+^ T cells, macrophage and neutrophil, which showed 53, 84, 71 and 53% reductions, respectively, relative to the amount of cells in the ears of mice from the SGE-1X. In contrast, the amount of CD8^+^ T cells that migrated to the ear of the SGE-3X group was 70% higher than the SGE-1X group (Figure 2B). Regarding to dendritic cells, there was no difference among all groups analyzed (Figure 2D). Therefore, pre-exposure of saliva leads to changes in the pattern of leukocyte migration to the site of inoculation.

### The effect of different SGE doses on the course of *L. braziliensis* infection

**Figure 3:**
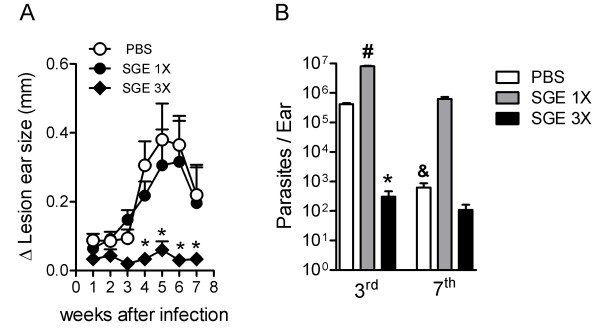
**Effect of *L. longipalpis *SGE on the course of *L. braziliensis *infection.** BALB/c mice inoculated i.d. once (SGE-1X) or three times (SGE-3X) with *Lutzomyia longipalpis* SGE or with PBS (control) were challenged with 10^5^*L. braziliensis* stationary phase promastigotes forms. The course of infection was monitored weekly by measuring the ear lesion size with a metric caliper. In **A***,* the lesion size was determined by the difference between the infected ear and the opposite uninfected ear given in millimeters (mm) (n = 5 mice per group). Data represent the mean ± SEM and are representative of two independent experiments. ^#^*P* < 0.05 compared with PBS. ^*^P < 0.05 compared with the SGE1-X or SGE-3X group. Ear parasitic burden at the 3rd and 7th week post-infection were determined by a limiting-dilution assay **(B).** The data shown represent the mean ± SEM of two independent experiments, and each experiment was performed with five mice per group (n = 5). ^#^*P* < 0.05 compared with PBS group. ^&^*P* < 0.05 compared with PBS group. ^*^P < 0.05 compared with the SGE-1X group.

Next, we evaluated whether pre-exposure to saliva interferes with the course of *L. braziliensis* infections. To this end, 1 × 10^5^*L. braziliensis* stationary phase promastigote forms suspended in PBS or SGE were inoculated into BALB/c mice ear pretreated with PBS-2X or SGE-2X. The development of the lesion was monitored weekly by measuring the diameter of the infected ear with a vernier caliper and comparing it with the non-infected ear on the same mouse. Mice challenged with the parasite in the presence of SGE-1X or PBS showed an increased in the lesion beginning on the 3rd week and continued to progress until the 5th week of infection (p < 0.05) (Figure 3A). After the 5th week, we observed a decrease in the ear size until the 7th week. Despite similar rates of edema in both groups (SGE-1X and PBS), mice that received SGE-1X showed higher parasite titers in the ear at the 3rd and 7th week post-infection when compared with mice inoculated with parasites in PBS (Figure 3B). Conversely, mice pretreated with saliva 2X and challenged with SGE plus parasite, referred to as SGE-3X, did not exhibit edema until the 7th week of infection. Furthermore, significantly lower numbers of parasites were detected on the 3rd and 7th week post-infection in mice that received SGE-3X when compared with mice that received parasite in SGE-1X (Figure 3B). In summary, our results are consistent with previous studies, which have shown that pre-exposure to saliva results in the protection against infection.

**Figure 4:**
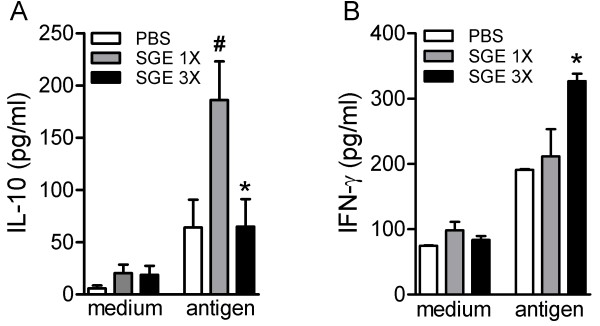
**Cytokine production by the draining lymph nodes after different inoculums of SGE.** BALB/c mice inoculated i.d. once (SGE-1X ) or three times (SGE-3X) with *Lutzomyia longipalpis* SGE or with PBS (control) were challenged with 10^5^*L. braziliensis* stationary phase promastigote forms. At the end of the 7th week post-infection, draining lymph node cells were harvested and restimulated *in vitro* with *L. braziliensis* antigen (5 μg/ml) or medium for 72 h. IL-10 **(A)** and IFN-γ **(B)** levels in the supernatant were determined by ELISA assay. The results are expressed as the mean ± SEM of at least two independent experiments using four to five mice per group (n = 4-5 mice per group). ^#^*P* < 0.05 compared with medium-only stimulus. ^*^P < 0.05 compared with the SGE-1X group.

Furthermore, we analyzed the ability of the draining lymph node cells from SGE-1X-, SGE-3X- or PBS-inoculated mice at the 7th week post-infection to produce IL-10 and IFN-γ in an attempt to understand the mechanism by which saliva exacerbates or protect mice against parasitic infection. Our results showed that the total lymph node cells from SGE-1X-inoculated mice produced more IL-10 after stimulation *in vitro* with parasitic antigen relative to mice inoculated with PBS or SGE-3X (Figure 4A). On the contrary, SGE-3X-treated mice produced significantly increased levels of IFN-γ when compared with the other groups of infected mice (Figure 4B).

**Figure 5:**
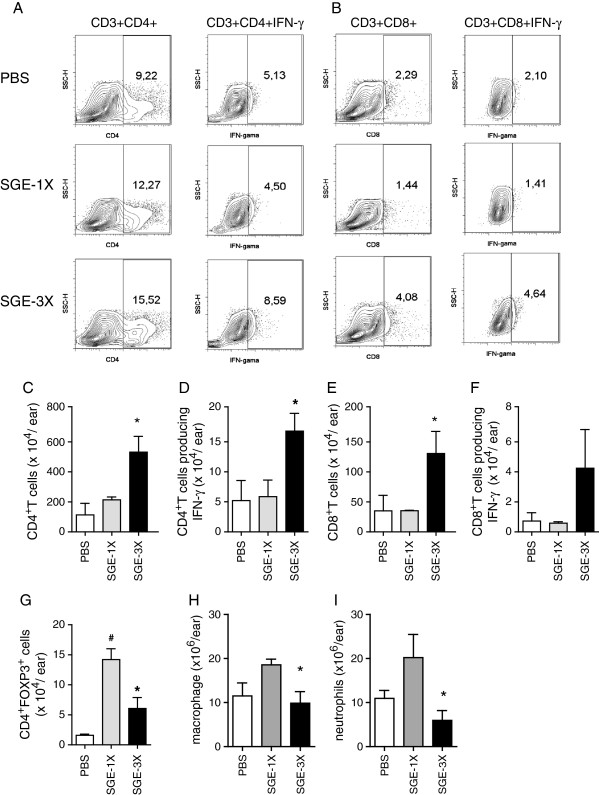
**Inflammatory profile during *L. braziliensis *infection after co-inoculation or pre-sensitization with saliva.** BALB/c mice inoculated i.d. once (SGE-1X) or three times (SGE-3X) with *Lutzomyia longipalpis* SGE or with PBS (control) were challenged with 10^5^*L. braziliensis* stationary phase promastigote forms. At the end of 7th week post-infection, ears were harvested, processed and inflammatory leucocytes were sorted using specific antibodies. For intracellular cytokines, the cells were *in vitro* re-stimulated with lived parasites. Dot plots represent the percentages of CD4^+^CD3^+^ and CD4^+^IFN-γ^+^ cells (**A**–left panel), CD8^+^CD3^+^ and CD8^+^IFN-γ^+^ cells (**B**–right panel). Total number of CD4^+^ T cells **(C)** and CD4^+^IFN-γ^+^ cells **(D)** or CD8^+^ T cells **(E)** and CD8^+^IFN-γ^+^ cells **(F),** CD4^+^FOXP3^+^ cells **(G),** macrophages **(H)** and neutrophils **(I)** within the ears were identified by flow cytometry. Data represent the mean ± SEM and are representative of two different experiments (n = 4). ^#^*P* < 0.05 compared with PBS. ^*^P < 0.05 compared with the SGE-1X group.

The cells that migrated to the site of parasite inoculation were identified by flow cytometry. As shown in Figure 5, *L. braziliensis* infection induced the recruitment of T lymphocytes such as CD4^+^ T and CD8^+^ T. Likewise, both populations were detected in the ears of SGE-1X-inoculated mice. In addition, similar numbers of CD4^+^ T cells and CD8^+^ T cells producing IFN-γ ex vivo were found in both the SGE-1X and the PBS group. By comparison, the leukocyte influx was altered in the ears of SGE-3X-inoculated mice. SGE-3X markedly increased the numbers of CD4^+^ T and CD8^+^ T cells by two- to three-fold when compared with the SGE-1X group. Furthermore, the production of IFN-γ by both T lymphocyte populations was higher in the SGE-3X group.

*L. braziliensis* infection induced the migration of CD4^+^FOXP3^+^ regulatory T cells to the ear lesion (Figure 5G). However, SGE-1X treatment enhanced the number of CD4^+^FOXP3^+^ cells by three- to four-fold in the site of infection. Furthermore, in contrast with aforementioned cells, the number of CD4^+^FOXP3^+^ T cells was significantly reduced by one- to two-fold in the SGE-3X group.

Our results also shown that, despite of SGE-1X presented the enhancement of neutrophil and macrophage, in the SGE-3X group both cell population was reduced. These reductions were, in average, 47% to macrophage (Figure 5H) and 48% to neutrophil (Figure 5I). These results therefore suggest that different saliva inoculums alters the inflammatory cell and cytokine composition at the site of parasite inoculation, and modulate the immune response during *L. braziliensis* infection.

### The protective effect of saliva is mediated by IFN-γ release

Because SGE-3X treatment protected the mice from parasitic infection (Figure 3) and induced significant production of IFN-γ (Figure 4B) by increasing the emigration of CD4^+^ T cells and CD8^+^ T cells (Figure 5), we further investigated the impact of IFN-γ production on resistance against *L. braziliensis* infection.

BALB/c mice sensitized with three treatments of saliva (SGE-3X) were depleted of IFN-γ by treatment with anti-IFN-γ mAb (R46A2 clone) and then were challenged with the parasite. As a control group, mice were also treated with a non-relevant IgG antibody.

**Figure 6:**
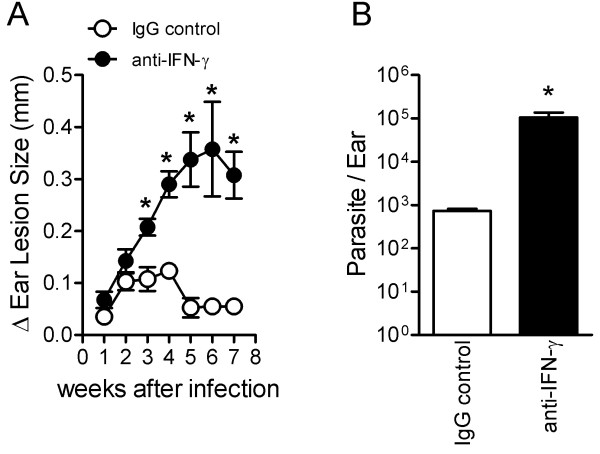
**Effects of *in vivo *depletion of IFN-γ on SGE-3X-inoculated mice.** BALB/c mice inoculated i.d. three times (SGE-3X) with *Lutzomyia longipalpis* SGE were challenged with 10^5^*L. braziliensis* stationary phase promastigote forms. Animals were treated with normal rat IgG or rat anti-IFN-γ. The course of infection was monitored weekly by measuring the ear lesion size with a metric caliper. In **A**, the lesion size was determined by the difference between the infected ear and the opposite uninfected ear in millimeters (mm) of at least five mice per group. Data represent the mean ± SEM and are representative of two independent experiments. *P < 0.05 compared with IgG control group. Ear **(B)** parasite burdens were determined at the 4th week post-infection via a limiting-dilution assay**.** The data shown are the mean ± SEM of two independent experiments, each performed with five mice per group. #*P* < 0.05 compared with PBS. *P < 0.05 compared with the SGE-1X group.

As shown in Figure 6A, SGE-3X mice treated with IgG control antibody developed minor edema that rapidly decreased with healing skin. Moreover, low parasitic titers were detected in this group (Figure 6B). Conversely, depletion of INF-γ reduced the protective effect of saliva on the course of *L. braziliensis* infection leading to a significant increase in the ear lesion size (p < 0.05) beginning at 3rd week of infection and persisting throughout the period of analysis (Figure 6A). Importantly, mAb anti-IFN-γ treatment also resulted in an increase in parasitic load at the inoculation site.

## Discussion

In this study, we reported that the dual effect of salivary gland extract (SGE) saliva from *Lutzomyia longipalpis* on the susceptibility or resistance of mice to *Leishmania braziliensis* infection is characterized by distinct changes in cellular immunity due to coinoculation or pre-exposure to saliva. Defining the nature of the inflammatory leucocytes that emigrate after saliva injection may help in the understanding of *Leishmania* infection biology and, therefore, may help in the development of new vaccine approaches that effectively protect the host against parasitic infection.

Studies have reported that immunization of mice with Phlebotomine saliva confers upon the mice a protective phenotype against *Leishmania* sp., whereas parasite and saliva that is simultaneously co-injected exacerbates infection, suggesting that immune responses triggered by the Phlebotomine saliva could represent a critical step in the development of disease. In this study, we showed that SGE inoculated once (SGE-1X), representing a co-inoculation, associated with a marked recruitment of several leucocytes, and most leucocytes were of the macrophage and neutrophil lineage. Interestingly, pre-exposure to saliva (SGE inoculated three times – SGE-3X) completely changed the cellular infiltrate composition. SGE-3X itself or during infection drastically reduced the amount of neutrophils and macrophages that migrated into the inoculation site even in the absence of parasitic infection, suggesting that the blockage of neutrophils and macrophage recruitment to the inflammatory site by saliva immunization is important to induce immune protection against parasitic infection. In fact, recent studies have described that neutrophils recruited to the site of *Leishmania* infections internalize the parasite [26,27], and saliva enhances neutrophil migration to the site of infection [28]. Previous studies have also observed that parasite internalization delays the apoptosis of neutrophils and induces MIP-1β release, which recruits macrophages to the site of infection. The migrated macrophages ingest the infected apoptotic neutrophils, which stimulates the release of TGF-β and PGE_2_ and downregulates macrophage activation consequently contributing to *Leishmania* infection establishment [26,27]. Together, these findings suggest that the parasites use granulocytes as “Trojan horses” to attack the macrophages [26]. In this context, the inhibition of both neutrophils and macrophages by saliva pre-exposure as described in the present investigation may represent an additional mechanism to explain the ability of Phlebotomine saliva pre-inoculation to protect mice against *Leishmania* infection.

Stressing the relevance of our finding, we demonstrated for the first time that Phlebotomine saliva increases regulatory T cell (Treg) recruitment to the lesion site. We demonstrated that inoculation of saliva once (SGE-1X) in the absence of parasites induces the recruitment of high numbers of CD4^+^CD25^+^ cells that, although being commonly accepted phenotype of Tregs also could be related to activated cells. Accordingly, parasites co-inoculated with saliva (SGE-1X) caused an increase in the recruitment of CD4^+^Foxp3^+^ cells to the infection site, suggesting that saliva of *L. longipalpis* increases Tregs during the infection. Despite the fact that the parasite alone is able to induce Treg migration, saliva strengthens this migration, which maintains the persistence of the parasite in the chronic phase of infection, and suggests that the recruitment of Tregs by the saliva may contribute to the infectivity of *Leishmania*. In fact, increased numbers of parasites at later time points were observed in the ears of mice co-inoculated with saliva and parasite, which corresponds to the point at which the disease becomes resolved and the parasitic burden decreases in the ears of mice infected with parasite only. Previous studies have also demonstrated that during infection with *L. major*, the persistence of the pathogen within the skin of *L. major*-resistant mice is controlled by an endogenous population of Treg cells that act to suppress the immune response against *L. major*. Treg cells are involved in maintaining the latency status of *Leishmania* infections and facilitate the survival of the parasite [29]. Our group reported that CD4^+^CD25^+^ T cells present in skin lesions of patients with cutaneous leishmaniasis display phenotypic and functional characteristics of natural Treg cells [30]. Thus, Treg cells induced by saliva play an important role in modulating the immune response during *Leishmania* infections. Notably, CD4^+^Foxp3^+^ cells were at normal levels in the ears of mice pre-sensitized to saliva (SGE-3X), which may have contributed to the control of *Leishmania* growth.

Our analyses of cytokine production further support the idea that SGE affects the inflammatory cell influx. Interestingly, our data show that *in vitro* stimulation of draining lymph node cells from SGE-1X mice with parasitic antigens results in higher levels of IL-10, whereas the IL-10 level in SGE-3X-derived draining lymph nodes cell cultures remained unchanged. Whereas the production of IL-10 was unchanged in the SGE-3X mice, IFN-γ production increased in the supernatant of SGE-3X lymph node-derived cell cultures, indicating that the inhibition of IL-10 in the SGE-3X mice may have resulted in better control of *Leishmania* infection. In fact, the severity of disease represented by the lesion size and parasitic burden was not observed in mice pre-sensitized with saliva (SGE-3X).

IL-10 is an anti-inflammatory cytokine produced by several cell types including macrophages, neutrophils and Treg cells, and IL-10 displays diverse immunomodulatory functions [31,32]. In regard to leishmaniasis, IL-10 inhibits cytokine production by T cells (e.g., IL-2), monocytes/macrophages and dendritic cells (e.g., IL-1α and IL-1β, IL-6, IL-8, IL-12, TNF-α, and granulocyte-macrophage colony-stimulating factor) as well as the production of NO and H_2_O_2_ ultimately favoring parasitic survival [32,33]. The hypothesis that IL-10 induced by saliva is involved in disease progression during *Leishmania* infection is supported by a significant enhancement in lesion development and parasitic burden in mice that were co-inoculated with saliva and parasites. The increase in IL-10 production has been reported in treatment with other Phlebotomine saliva sources. In previous studies, we demonstrated that the saliva from the Old World species Phlebotomines *P. papatasi* and *P. duboscqi* act mainly on dendritic cells and induce the production of IL-10 by a mechanism dependent of PGE_2_. In turn, PGE_2_ acts in an autocrine manner to reduce the antigen-presenting ability of DCs [13]. Previous studies have also shown *in vitro* and *in vivo* examples of *Lutzomyia longipalpis* saliva promotes inducing IL-10 production by macrophages and T cells, which exacerbates *Leishmania* infection [34]. Moreover, the genetic ablation of IL-10 prevents the detrimental effect of SGE on *Leishmania major* and *L. amazonensis* infections.

The reduced ability of SGE-3X- inoculated mice to produce IL-10 may be associated with an increase in IFN-γ production. Consistently, the depletion of IFN-γ using IFN-γ-neutralizing monoclonal antibody reduced the protective profile of saliva upon *Leishmania* disease. Despite the significant increase in CD8^+^ T cells in the ears of mice that were pre-inoculated with saliva three times (SGE-3X), our evidence suggests that CD4^+^ T cells and CD8^+^ T cells contributed to the increased *ex vivo* production of IFN-γ during *Leishmania* infection. Although the present study is insufficient to declare the mechanism by which the immune cells mediate the control of *Leishmania* growth, we believe that the presence of lymphocytes producing IFN-γ at the site of inflammation may induce the direct killing of *L. braziliensis* by nitric oxide (NO)-dependent mechanisms. This effect could be mediated by proteins presents into saliva that are uptake by antigen- presenting cells and prime naïve CD4^+^T cell and CD8^+^T cells. When the mice are challenged with parasite in the presence of saliva, it triggers a rapid T cells activation and production of IFN-γ. Thus, there is a cross-reactivity of the immune response induced by salivary proteins against *Leishmania braziliensis*. This hypothesis has been validated in models with salivary proteins. PpSP15 protein derived from *Phlebotomus papatasii* provided protective immune response against *L. major* when the parasite was co-inoculated with *P. papatasi* SGE by the induction of DTH response [16]. Likewise, the immunization of mice with proteins from *Lutzomyia longipalpis*, LJM11 and LJM19 induced the strong DTH and conferred the protective effect against different species of Leishmania (*L. major*, *L. infantum* and *L. braziliensis*) when the mice were challenged with parasite and SGE [35-39]. Interestingly, such responses were similar with that previously obtained using a natural sensitization with bites of uninfected sand fly [15].

Several pieces of evidence have shown that Phlebotomine saliva enhances the infectivity of many different *Leishmania* species, which can be attributed to numerous substances within the saliva that harbor pharmacological properties that induce vasodilatation, anticoagulation, anti-inflammation and immunomodulation. Thus, the active salivary constituents could serve as a prototype for the development of vaccines to control pathogen transmission. Our group is currently working on the isolation of compounds within the saliva of several blood-feeding arthropods, including Phlebotomine vectors. We recently identified adenosine (ADO) and adenosine monophosphate (AMP) as major immunomodulatory compounds present within the Old World sand fly species *Phlebotomus papatasii,* which protected mice from extreme inflammatory insults [40]. Salivary protein (SP)-15 is also present in *P. papatasi*, and SP-15 provides a protective effect against *Leishmania major* infection through an IFN-γ-dependent mechanism [16]. In the present study, neither ADO and AMP nor SP-15 is involved in the effect of SGE on *Leishmania* infection because they are not found in *Lutzomyia longipalpis* saliva. Maxadilan (MAX) is a potent vasodilator present in *L. longipalpis* saliva that exacerbates *Leishmania sp*. infection. Mice vaccinated with recombinant MAX were markedly protected from *Leishmania* infection, and this protective effect was associated with an increase in CD4^+^ T cells, IFN-γ and NO [14]. Presently, we cannot dismiss the possibility that the protective effect of SGE-3X could be mediated by MAX; however, several other proteins (LJM17 and LJM11 in human and LJM17, LJM11, LJL13, LJL23 and LJL143 in dogs) from *L. longipalpis* saliva have been identified [41], suggesting that intensive efforts are required for the identification of salivary compounds responsible for the protective effect of sand fly saliva on leishmaniasis.

## Conclusion

In summary, the present study provides strong evidence that different *Lutzomyia longipalpis* saliva inoculation schemes may skew the initial cellular responses, which is reflected by parasitic survival or host resistance to infection. Thus, we believe that comprehending the effects of sand fly saliva on the host immune response induced by saliva may help in the generation of new vaccine strategies that can block the effects of saliva and prevent *Leishmania* establishment in the host.

## Abbreviations

SGE-1X: Salivary gland extract inoculated once; SGE-3X: Salivary gland extract inoculated three times.

## Competing interest

The authors declare that they have no competing interest.

## Author contributions

Conceived and designed the experiments: VC and JSS. Performed the experiments: VC and DLC. Analyzed the data: VC and JSS. Contributed reagents/materials/analysis tools: CIB, AMB, MB, FQC and JSS. Wrote the paper: VC and JSS. Revised the paper DLC, CIB, AMB, MB and FQC. All authors read and approved the final manuscript.
